# Discovery of Four Novel Circular Single-Stranded DNA Viruses in Fungus-Farming Termites

**DOI:** 10.1128/genomeA.00318-18

**Published:** 2018-04-26

**Authors:** Mason Kerr, Karyna Rosario, Christopher C. M. Baker, Mya Breitbart

**Affiliations:** a College of Marine Science, University of South Florida, Saint Petersburg, Florida, USA; b Department of Ecology and Evolutionary Biology, Princeton University, Princeton, New Jersey, USA

## Abstract

Here, we describe four novel circular single-stranded DNA viruses discovered in fungus-farming termites (Odontotermes sp.). The viruses, named termite-associated circular virus 1 (TaCV-1) through TaCV-4, are most similar to members of the family Genomoviridae and were widely detected in African termite mounds.

## GENOME ANNOUNCEMENT

Fungus-farming termites from the genus Odontotermes build extensive mounds that can enhance ecosystem productivity (1). These social insects have symbiotic fungi and bacteria that digest foraged plant material and serve as nutritional sources for the termites (2–4). Although several studies have characterized bacterial and fungal communities associated with fungus-farming termites (5, 6), viruses associated with these unique agricultural systems have not been investigated. Here, we describe four novel circular single-stranded DNA (ssDNA) viruses discovered in Odontotermes sp. termites.

Termites were collected opportunistically from individual mounds located at the Mpala Research Center in Kenya and catalogued based on caste, mainly workers and soldiers, with nymphs collected less commonly. Castes from each mound were screened for the presence of ssDNA viral genomes encoding a replication-associated protein (Rep) following methods previously used for the discovery of circular ssDNA viruses in dragonflies (7). Briefly, virus-like particles were partially purified from each sample by homogenization and filtration through a 0.45-µm Sterivex filter (Millipore). DNA was extracted from the filtrate using the MinElute virus spin kit (Qiagen) and amplified through rolling circle amplification (RCA). RCA products were then digested with restriction enzymes in separate reactions, and digestion products between 1 and 4 kb were cloned using the CloneJET PCR cloning kit (Thermo Scientific) and sequenced with vector primers. Sequences with significant matches to known circular, Rep-encoding ssDNA (CRESS DNA) viruses were used to design back-to-back primers to obtain complete genomes through inverse PCR.

Four CRESS DNA viruses, named termite-associated circular virus 1 (TaCV-1) through TaCV-4, were detected in workers. BLAST searches in GenBank and the genome organization of TaCVs indicate that these viruses belong to the family Genomoviridae. Similar to genomoviruses, the TaCV genomes range from 2,155 to 2,222 nucleotides (nt) in length and contain two major open reading frames (ORFs) encoding a Rep and a capsid protein. The Rep ORF for TaCV-2, TaCV-3, and TaCV-4 is interrupted by an intron. The four TaCVs share <70% genome-wide pairwise identity with each other and previously described genomoviruses, indicating that each TaCV represents a novel species within the family Genomoviridae (8). Based on the Rep ORF, TaCV-1, TaCV-2, and TaCV-4 are most closely related to genomoviruses identified in animal feces, insects, and sewage, respectively (9–11), while TaCV-3 is most similar to a plant-infecting mastrevirus (12).

PCR assays revealed the presence of one or more TaCVs in 23% of the 47 mounds tested, including mounds separated by 11 km. TaCV-1 was the most prevalent since it was detected in 8 of the 11 TaCV-positive mounds, compared to TaCV-2, TaCV-3, and TaCV-4, which were found in 2, 3, and 1 mound, respectively. Notably, TaCVs were only detected in the worker caste. Since termite workers ingest fungi and maintain the fungal gardens (13, 14), and genomoviruses have been identified in both fungi and insects (15, 16), it is difficult to predict the host of the novel TaCVs described here. Nevertheless, the widespread detection of TaCVs in termite mounds suggests that these ssDNA viruses may play a role in the ecology of fungus-farming termite systems.

### Accession number(s).

The genome sequences of termite-associated circular virus 1 (TaCV-1) through TaCV-4 have been deposited in GenBank under the accession numbers MG917674 to MG917677.
